# Supplementary material for: The role of anharmonic phonons in under-barrier spin relaxation of single molecule magnets
Source: Nat Commun. 2017 Mar 6;8:14620. doi: 10.1038/ncomms14620 (PMC5343443; doi:10.1038/ncomms14620)
Supplement: Supplementary Information — Supplementary Figures, Supplementary Tables, Supplementary Notes and Supplementary References. [file ncomms14620-s1.pdf]

SUPPLEMENTARY NOTE 1. STOCHASTIC MODELING OF PHONONS LINE-WIDTH.

The diagonal elements of the spin reduced density matrix can be expressed through the second order time convolution less master equation

$$\frac{d\rho^{\mathrm{S}}_{aa}(t)}{dt} = \frac{2}{\hbar^2}\sum_{c\alpha}\Big\{-\sum_{j}\delta_{ac}V^{\alpha}_{aj}V^{\alpha}_{jc}\mathcal{G}(t,\omega_{ja},\omega_\alpha) + |V^{\alpha}_{ac}|^2\mathcal{G}(t,\omega_{ca},\omega_\alpha)\Big\}\rho^{\mathrm{S}}_{cc}(t)\ , \tag{1}$$

provided the validity of the weak coupling regime for the spin and the phonons degrees of freedom (Born approximation). Under this approximation, Supplementary Eq. 1 can be used to describe the non-markovian dynamics of the spin open-system. The quantity $\mathcal{G}(t,\omega_{ij},\omega_\alpha)$ is the α-phonon green function calculated at the time t and it is defined as

$$\mathcal{G}(t,\omega,\omega_\alpha) = \int_0^t dt' e^{-i\omega t'}\mathrm{Tr}_{\mathrm{B}}\left[\hat{q}_\alpha(t)\hat{q}_\alpha(t-t')\rho^{\mathrm{eq}}_{\mathrm{B}}\right]\ , \tag{2}$$

where the subscript 'B' stands for all the degrees of freedom of the phonon bath and the operator $Tr_{\mathrm{B}}\left(...\hat{\rho}^{\mathrm{eq}}_{\mathrm{B}}\right)$ represents the ensemble equilibrium average over the bath. In Supplementary Eq. (2) $\hat{q}_\alpha = \frac{1}{\sqrt{2}}(\hat{a}^\dagger_\alpha + \hat{a}_\alpha)$ is the displacement operator for the α-th mode with $\hat{a}^\dagger_\alpha$ ($\hat{a}_\alpha$) being the creation (annihilation) operator for that mode of frequency ω_α, and $\hat{\rho}^{\mathrm{eq}}_{\mathrm{B}}$ is the equilibrium density matrix for the bath. $\mathcal{G}(t,\omega,\omega_\alpha)$ contains all the information on the bath dynamics, time and temperature dependence included. This mathematical object is usually studied perturbatively, where the harmonic approximation is used as the ground for the Dyson's equation and the anharmonic interactions between phonons represent a perturbation acting on the normal modes. The presence of anharmonic interactions makes the life-time of phonons finite and consequently the bath spectral shape broadened. In order to access this information about the bath, one requires calculating the anharmonic frequencies and the phonon self-energy [1]. In this work we have tackled the problem through a stochastic approach, which has the advantage of not requiring information about the single-phonon phonon-phonon interactions. Within this framework, the bath Hamiltonian is assumed to have an harmonic form, $\hat{H}_{\mathrm{ph}} = \sum_\alpha \hat{H}_{\mathrm{ph},\alpha} = \sum_\alpha \hbar\omega_\alpha(\hat{n}_\alpha + \frac{1}{2})$, and all the anharmonic features are introduced in a statistical way, according to the fact that the interaction of a mode with the bath (made by all the other modes) is the same interaction that drives it to the thermal equilibrium described by the canonical distribution. If a phonon is at thermal equilibrium, its energy will experience fluctuations and consequently, any eigenstate of $\hat{H}_{\mathrm{ph}}$ will have a finite life-time. Clearly there is an equivalence between the statistical picture and the mechanistic microscopic description of the phonon-bath system[2]. In order to bridge the statistical description with the microscopic one, the phonon Hamiltonian is considered to be time dependent with a time propagator $e^{-i\int_0^{t'} d\tau \hat{H}_{\mathrm{ph}}(\tau)}$. The expression for the time-dependent bath normal mode displacement operators is

$$\hat{q}(t) = e^{i\int_0^t d\tau \hat{H}_{\mathrm{ph}}(\tau)}\hat{q}e^{-i\int_0^t d\tau \hat{H}_{\mathrm{ph}}(\tau)}\ . \tag{3}$$

Therefore we have

$$\mathrm{Tr}_{\mathrm{B}}\left[\hat{q}_\alpha(t)\hat{q}_\alpha(t-t')\hat{\rho}^{\mathrm{eq}}_{\mathrm{B}}\right] = \tag{4}$$

$$\mathrm{Tr}_{\mathrm{B}}\left[e^{i\int_0^t d\tau \hat{H}_{\mathrm{ph}}(\tau)}\hat{q}_\alpha e^{-i\int_0^t d\tau \hat{H}_{\mathrm{ph}}(\tau)}e^{i\int_0^{t-t'} d\tau \hat{H}_{\mathrm{ph}}(\tau)}\hat{q}_\alpha e^{-i\int_0^{t-t'} d\tau \hat{H}_{\mathrm{ph}}(\tau)}\hat{\rho}^{\mathrm{eq}}_{\mathrm{B}}\right] = \tag{5}$$

$$\mathrm{Tr}_{\mathrm{B}}\left[e^{i\int_0^t d\tau \hat{H}_{\mathrm{ph}}(\tau)}(\hat{a}^\dagger_\alpha + \hat{a}_\alpha)e^{-i\int_0^t d\tau \hat{H}_{\mathrm{ph}}(\tau)}e^{i\int_0^{t-t'} d\tau \hat{H}_{\mathrm{ph}}(\tau)}(\hat{a}^\dagger_\alpha + \hat{a}_\alpha)e^{-i\int_0^{t-t'} d\tau \hat{H}_{\mathrm{ph}}(\tau)}\hat{\rho}^{\mathrm{eq}}_{\mathrm{B}}\right]\ . \tag{6}$$

The time dependence of $\hat{H}_{\mathrm{ph}}$ enters into ω_α that can be expressed as $\omega_\alpha(t) = \langle\omega_\alpha\rangle + \delta\omega_\alpha(t)$. According to the Born approximation ($\hat{\rho}_{\mathrm{B}}(t) \sim \hat{\rho}^{\mathrm{eq}}_{\mathrm{B}}$), we assume that the bath relaxes to equilibrium as its equilibrium fluctuations do, in a fluctuation-dissipation theorem fashion. Therefore, the phonons' energies will be considered randomly fluctuating around the microcanoical (NVE) $\langle\omega_\alpha\rangle$ frequencies with a gaussian distribution of amplitude $\Delta = \sqrt{\langle\delta E^2\rangle} = \sqrt{\langle E^2\rangle - \langle E\rangle^2}$, where all the mean values are taken at the canonical ensamble (NVT) equilibrium. As the time dependent part of the bath Hamiltonian is not due to its operator part we obtain $[\hat{H}_{\mathrm{ph}}(t), \hat{H}_{\mathrm{ph}}(t')] = 0$ and thus $e^{i\int_0^t d\tau \hat{H}_{\mathrm{ph}}(\tau)} = \prod_{n=0}^t e^{i\Delta\tau \hat{H}_{\mathrm{ph}}(\tau_n)}$, where all the exponents commute. According to this, it is then possible to demonstrate that

$$e^{i\int_0^t d\tau \hat{H}_{\rm ph}(\tau)} \hat{a}_\alpha^\dagger e^{-i\int_0^t d\tau \hat{H}_{\rm ph}(\tau)} = e^{i\int_0^t d\tau \omega_\alpha(\tau)} \hat{a}_\alpha^\dagger \tag{7}$$
$$e^{i\int_0^t d\tau \hat{H}_{\rm ph}(\tau)} \hat{a}_\alpha e^{-i\int_0^t d\tau \hat{H}_{\rm ph}(\tau)} = e^{-i\int_0^t d\tau \omega_\alpha(\tau)} \hat{a}_\alpha \,, \tag{8}$$

so that Supplementary Eq. (6) becomes

$$\mathrm{Tr_B}\Big[\hat{q}_\alpha(t)\hat{q}_\alpha(t-t')\hat{\rho}_{\rm B}^{\rm eq}\Big] = \mathrm{Tr_B}\Big[(e^{i\int_0^{t'} d\tau\omega_\alpha(\tau)}\hat{a}_\alpha^\dagger\hat{a}_\alpha + e^{-i\int_0^{t'} d\tau\omega_\alpha(\tau)}\hat{a}_\alpha\hat{a}_\alpha^\dagger)\hat{\rho}_{\rm B}^{\rm eq}\Big] \,. \tag{9}$$

Before proceeding further we should consider the relation

$$\mathrm{Tr_B}\Big[(e^{i\int_0^{t'} d\tau\omega_\alpha(\tau)}\hat{a}_\alpha^\dagger\hat{a}_\alpha + e^{-i\int_0^{t'} d\tau\omega_\alpha(\tau)}\hat{a}_\alpha\hat{a}_\alpha^\dagger)\hat{\rho}_{\rm B}^{\rm eq}\Big] =$$
$$\mathrm{Tr_B}\Big[e^{i\int_0^{t'} d\tau\omega_\alpha(\tau)}\hat{\rho}_{\rm B}^{\rm eq}\Big]\mathrm{Tr_B}\Big[\hat{a}_\alpha^\dagger\hat{a}_\alpha\hat{\rho}_{\rm B}^{\rm eq}\Big] + Tr_{\rm B}\Big[e^{-i\int_0^{t'} d\tau\omega_\alpha(\tau)}\hat{\rho}_{\rm B}^{\rm eq}\Big]\mathrm{Tr_B}\Big[\hat{a}_\alpha\hat{a}_\alpha^\dagger\hat{\rho}_{\rm B}^{\rm eq}\Big] \,. \tag{10}$$

Finally, according to Kubo's model [3, 4], we can write

$$\mathrm{Tr_B}\Big[e^{i\int_0^{t'}\omega_\alpha(\tau)}\hat{\rho}_{\rm B}^{\rm eq}\Big] = \langle e^{i(\langle\omega_\alpha\rangle + \int_0^{t'}\delta\omega_\alpha(\tau))}\rangle = e^{i\langle\omega_\alpha\rangle t'}\langle e^{i\int_0^{t'}\delta\omega_\alpha(\tau)}\rangle = e^{i\langle\omega_\alpha\rangle t'}F(t') \tag{11}$$

We can then expand $F(t')$ as a cumulant expansion of averages and take advantage of the Gaussian statistic of fluctuations

$$F(t') = \exp\Big[-\frac{1}{2}\int_0^{t'}\int_0^{t'} d\tau' d\tau''\langle\delta\omega(\tau')\delta\omega(\tau'')\rangle\Big] \,, \tag{12}$$

which, by making the substitution $\tau = \tau' - \tau''$, becomes

$$F(t') = \exp\Big[-\int_0^{t'} d\tau(t'-\tau)\langle\delta\omega(\tau)\delta\omega(0)\rangle\Big] \,. \tag{13}$$

Finally, by assuming that the fluctuation correlation function decays exponentially with a characteristic time τ_α *i.e.* $\langle\delta\omega(\tau)\delta\omega(0)\rangle = \Delta_\alpha^2\exp(-\tau/\tau_\alpha)$, we obtain

$$F(t) = \exp\Big[-\Delta_\alpha^2\tau_\alpha^2(\exp(-t/\tau_\alpha) + t/\tau_\alpha - 1)\Big] \,. \tag{14}$$

The Heisenberg principle offers a way to estimate the maximum limit of the τ_α according to a given energy fluctuation magnitude Δ_α, *i.e.* $\tau_\alpha = \Delta_\alpha^{-1}$. According to its statistical mechanics definition, Δ_α can be evaluated as

$$\Delta_\alpha^2 = \frac{\partial\langle H_{ph,\alpha}\rangle}{\partial\beta} = \frac{(\hbar\omega_\alpha)^2 e^{\beta\hbar\omega_\alpha}}{(e^{\beta\hbar\omega_\alpha}-1)^2} \,, \quad \beta = k_{\rm B}T \tag{15}$$

Supplementary Eq. (15) describes the temperature dependence of the phonon spectra line-width. At low temperature Δ converges to zero asymptotically and a $1/\beta$ linear divergence is observed for $\beta \to 0$. A similar temperature behaviour is experimentally observed through the determinations of the homogeneous contribution to the phonon line width [5–7]. Although Supplementary Eq. (15) qualitatively describes the correct observed behavior for Δ, a few discrepancies between this model and experiments exist. First of all, the experimental line-width approaches a finite value different from 0 for $T \to 0$. Moreover, this model treats on the same footing every modes while experimentally the magnitude of the Δ divergence depends on the anharmonicity of the mode considered. However, given its extreme simplicity, the proposed model represents an appealing way to introduce anharmonic contributions in the bath Green's function without any further requirements apart from harmonic modes calculation.

Finally, it is possible to define the phonon spectral shape according to the phonon Green's function

$$\mathcal{G}(t,\omega,\omega_\alpha) = \int_0^t dt' e^{-i\omega t'}\mathrm{Tr_B}\Big[\hat{q}_\alpha(t)\hat{q}_\alpha(t-t')\hat{\rho}_{\rm B}^{\rm eq}\Big] = \tag{16}$$
$$= \int_0^t dt'\big[e^{-i(\omega-\omega_\alpha)t'}F(t')\bar{n}_\alpha + e^{-i(\omega+\omega_\alpha)t'}F(t')(\bar{n}_\alpha+1)\big] \,, \tag{17}$$

where $\bar{n}_\alpha = \frac{1}{e^{\beta\hbar\omega_\alpha}-1}$ is the phonon occupation number according to the Bose-Einstein statistics. Supplementary Eq. 17 can be further simplified under the condition that the integrand function would decay on a faster time-scale than the one under which the spin life-time evolves (Markov approximation). Under this assumption, the decay of F(t) becomes purely exponential and the integration limits of Supplementary Eq. 17 can be replaced with $\pm\infty$ to obtain a time independent bath Green function $G(\omega, \omega_\alpha)$:

$$F(t) \rightarrow F(t) = \exp(-\Delta_\alpha t) \tag{18}$$

$$\mathcal{G}(t, \omega, \omega_\alpha) \rightarrow G(\omega, \omega_\alpha) = \int_{-\infty}^{\infty} dt' e^{-i\omega t'} \mathrm{Tr_B}\left[\hat{q}_\alpha(t)\hat{q}_\alpha(t-t')\rho_\mathrm{B}^{\mathrm{eq}}\right] = \tag{19}$$

$$= \int_{-\infty}^{\infty} dt' \left[e^{-i(\omega-\omega_\alpha)t'} e^{-\Delta_\alpha t'} \bar{n}_\alpha + e^{-i(\omega+\omega_\alpha)t'} e^{-\Delta_\alpha t'} (\bar{n}_\alpha + 1)\right] = \tag{20}$$

$$= \frac{\Delta_\alpha}{\Delta_\alpha^2 + (\omega - \omega_\alpha)^2} \bar{n}_\alpha + \frac{\Delta_\alpha}{\Delta_\alpha^2 + (\omega + \omega_\alpha)^2} (\bar{n}_\alpha + 1) \,, \tag{21}$$

By using Δ_α as defined in Supplementary Eq. (15), the life-time of the normal mode ω=36 cm^{-1} is in the ns - ps range for temperatures in the $2 - 10$ K interval. This is orders of magnitude shorter than the spin relaxation time observed for $[(tpa^{Ph})\mathrm{Fe}]^-$ (ms - μs), demonstrating that Markov's approximation is valid for the dynamics discussed in the main text of the paper. When the Markov's condition is not fulfilled, one has then to replace Supplementary Eq. (21) with the more general Supplementary Eq. (17).

SUPPLEMENTARY NOTE 2. $S = 1$ MODEL DYNAMICS.

The three-level $S = 1$ model has been discussed in the main text and it is defined by the following energy ladder: $E_2 > E_1 \sim E_0$, i.e. $E_2 - E_1 \sim E_2 - E_0 = U_0$ and $E_1 - E_0 \sim 0$. As discussed in the main text, we assume that there is only one phonon able to directly interact with the spin system.

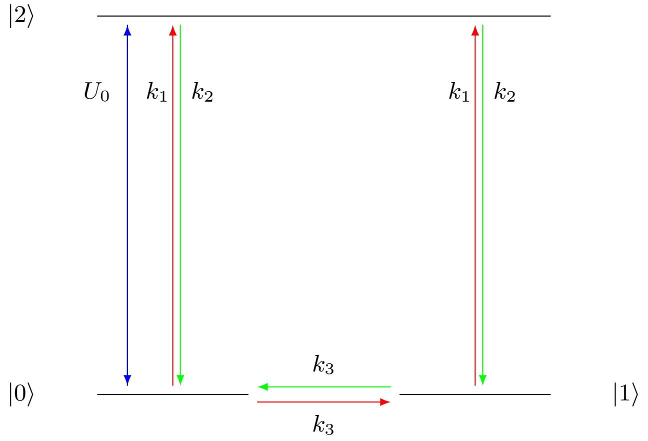

Supplementary Figure 1. **Energy level diagram for the $S = 1$ one-phonon model.** The blue line indicates the zero-field splitting energy, the red lines represent the population transfers due to one-phonon absorption and the green lines represent population transfers due to one-phonon emission.

The eigenstates populations p_n $(n = 0, 1, 2)$ evolve in time according to the kinetic equations

$$\begin{cases} \frac{dp_0}{dt} = -k_1 p_0 + k_2 p_2 - k_3 p_0 + k_3 p_1 \\ \frac{dp_1}{dt} = -k_3 p_1 + k_3 p_0 - k_1 p_1 + k_1 p_2 \\ \frac{dp_2}{dt} = -2k_2 p_2 + k_1 p_0 + k_1 p_1 \end{cases} \quad (22)$$

If one assumes an identical spin-phonon coupling strength V, for all the transitions, the various kinetic constants are defined as

$$k_1 = Vn(\hbar\omega)G(U_0, \hbar\omega)\ , \quad (23)$$

$$k_2 = V[n(\hbar\omega) + 1]G(-U_0, \hbar\omega)\ , \quad (24)$$

$$k_3 = V[2n(\hbar\omega) + 1]G(\delta, \hbar\omega)\ . \quad (25)$$

The kinetic constants k_3, associated to the transitions between the quasi-degenerate lower-lying states, $|0\rangle \rightarrow |1\rangle$ and $|1\rangle \rightarrow |0\rangle$, have been set to $V[2n(\hbar\omega) + 1]G(\delta, \hbar\omega)$. This is because of the assumption of $E_1 - E_0 = \delta \sim 0$ and the fact that the phonon has a frequency $\omega > 0$ and a finite line-width. In this scenario the transition between the two almost degenerate states is induced by the tail of the phonon distribution at an energy ~ 0.

If we now take the quantisation axis along the direction of an external magnetic field, or equivalently with the z-direction oriented in order to reduce the rhombic terms in the spin Hamiltonian, the magnetisation will be in good approximation $M_z(t) = p_0 - p_1$. Thus the dynamics equation for M_z can be written by subtracting the first and the second equation in (22), namely

$$\frac{dM_z(t)}{dt} = \frac{d(p_0 - p_1)}{dt} = -(2k_3 + k_1)(p_0 - p_1) = -(2k_3 + k_1)M_z(t)\ . \quad (26)$$

From Supplementary Eq. (26) it appears clear that $M_z(t)$ decays exponentially in time with the decay coefficient determined by the two kinetic constants k_3 and k_1. In order to understand the nature of the relaxation processes related to these two constants it is necessary to obtain eigenvectors and eigenvalues of the entire system of equations (22). However, instead of solving the Eqs. (22), it is more instructive to separate the problem into the direct intra ground-state doublet population transfer and the excited state mechanism (Orbach's mechanism). This is done, respectively, by forcing $k_1 = k_2 = 0$ and $k_3 = 0$.

Case 1. The solutions of the system (22) for the $k_1 = k_2 = 0$ case are

$$\begin{aligned} p_0(t) = &\ (p_0(0) - p_0(\infty))e^{-2k_3t} + p_0(\infty)\ , \\ p_1(t) = &\ p_1(\infty)(1 - e^{-2k_3t})\ , \end{aligned} \tag{27}$$

and thus

$$M_z(t) \propto [(p_0(0) - p_0(\infty) + p_1(\infty)]e^{-2k_3t} + p_0(\infty) - p_1(\infty)\ . \tag{28}$$

Under these conditions the magnetisation vector relaxes to its equilibrium value with a relaxation time $\tau = \frac{1}{2k_3}$. In the anharmonic case, *i.e.* assuming Δ as form Supplementary Eq.15, and in the $\hbar\omega \gg k_\mathrm{B}T \gg \delta$ limit we have

$$k_3 = V\frac{\Delta}{\Delta^2 + (\delta - \hbar\omega)^2} \sim V\frac{\Delta}{\Delta^2 + \hbar\omega^2} \sim \frac{V}{\hbar\omega}e^{-\frac{\beta\hbar\omega}{2}}\ , \tag{29}$$

and so the relaxation time follows an Arrhenius like-temperature dependence

$$\tau_{Dir} = \tau_0 e^{\beta U_\mathrm{eff}}\ , \qquad \tau_0 = \frac{\hbar\omega}{V}\ , \qquad U_\mathrm{eff} = \frac{\hbar\omega}{2}\ . \tag{30}$$

In the $k_\mathrm{B}T \gg \hbar\omega$ case we have

$$k_3 = V\frac{1}{e^{\beta\hbar\omega} - 1}\frac{\Delta}{\Delta^2 + (\delta - \hbar\omega)^2} = \frac{V}{\hbar\omega}\frac{e^{\frac{\beta\hbar\omega}{2}}}{(e^{\beta\hbar\omega} - 1)^2 + e^{\beta\hbar\omega}} \sim \frac{V}{\hbar\omega}\left(1 - \frac{1}{2}\beta\hbar\omega\right) \tag{31}$$

In the limit $\beta \to 0$, $\tau_{Dir} \sim \frac{\hbar\omega}{V}[1 - \frac{1}{2}\beta\hbar\omega)]^{-1} \sim \frac{\hbar\omega}{V}(1 + \frac{1}{2}\beta\hbar\omega)$.

In the harmonic case $G(\hbar\omega, \delta) = \delta(\hbar\omega - \delta)$ and therefore the relaxation would proceed only when a very-low-energy phonon is available to couple with the spin system. In this situation the most interesting case is $k_\mathrm{B}T \gg \delta$ and under these assumptions the population transfer rate reads

$$k_3 = V[2\hat{n}(\hbar\omega) + 1]\delta(\hbar\omega - \delta) = \frac{2V}{e^{\beta\delta} - 1} \sim \frac{2V}{\beta\delta}\ , \tag{32}$$

where the linear dependency of the direct relaxation mechanism with respect to T is recovered, i.e.

$$\tau_\mathrm{Dir} = \frac{\beta\delta}{2V}\ . \tag{33}$$

Case 2. When $k_3 = 0$, a situation describing a pure Orbach's relaxation mechanism, we have two eigenvalues beside the null one: k_1 and $2k_2 + k_1$. The eigenvector corresponding to k_1 is the same as for the case $k_1 = k_2 = 0$ just discussed and it corresponds to a population transfer from $|0\rangle$ to $|1\rangle$ or *vice versa*:

$$\begin{pmatrix} p_0 \\ p_1 \\ p_2 \end{pmatrix} = \begin{pmatrix} 1 \\ -1 \\ 0 \end{pmatrix} \tag{34}$$

Interestingly, this process, which introduces the k_1 kinetic constant in the magnetisation decay profile, does not transfer population to the excited states, but involve a virtually instantaneous excitation and emission of the system. It must be noted that even though the population of the excited state $|2\rangle$ is not involved in this process, the presence of this state is indirectly accounted for in the definition of the kinetic transfer rate constant k_1. The effect on the system of the eigenvalue $2k_2 + k_1$ is different from the former one and it corresponds to an eigenvector of the form

$$\begin{pmatrix} p_0 \\ p_1 \\ p_2 \end{pmatrix} = \begin{pmatrix} 1/\sqrt{6} \\ 1/\sqrt{6} \\ -2/\sqrt{6} \end{pmatrix} \tag{35}$$

This kind of eigenvector does not produce relaxation but merely adjust the population of the excited state to its equilibrium value and indeed it does not appear in the $M_z(t)$ rate equation (26).

Focusing on the sole through excited state mechanism, the transition rate is

$$k_1 = V\frac{1}{e^{\beta\hbar\omega} - 1}\frac{\Delta}{\Delta^2 + (U_0 - \hbar\omega)^2} = \frac{V}{\hbar\omega}e^{\frac{\beta\hbar\omega}{2}}\frac{1}{e^{\beta\hbar\omega} + \frac{(U_0 - \hbar\omega)^2}{(\hbar\omega)^2}(e^{\beta\hbar\omega} - 1)^2}\ , \tag{36}$$

which, in the resonance condition, becomes

$$k = \frac{V}{\hbar\omega} e^{-\frac{\beta\hbar\omega}{2}} , \tag{37}$$

for all temperatures. However, in the low-T regime, the general expression (36) implies a relaxation time constant

$$\tau_{\text{Orb}} = \frac{\hbar\omega}{V} \left[e^{\frac{\beta\hbar\omega}{2}} + \frac{(U_0 - \hbar\omega)^2}{(\hbar\omega)^2} e^{\frac{3}{2}\beta\hbar\omega} \right] . \tag{38}$$

It must be noted that when taking the definition of the phonon life-time of Supplementary Eq. (15) we are implicitly assuming that the magnitude of the phonon frequency sets the temperature at which the anharmonic effects become visible. Due to this feature of the model the phonon damping and its frequency are correlated quantities and it is no longer possible to recover the limit of zero damping without changing the harmonic features of the vibrational mode. For this reason the harmonic limit cannot be recovered from Supplementary Eq. (36) but must be taken before substituting Supplementary Eq. (15) in the definition of k_1. With this precaution, the population transfer rate in the harmonic limit is

$$k_1 = V\hat{n}(\hbar\omega)\delta(\hbar\omega - U_0) . \tag{39}$$

As for the direct mechanism, the relaxation in the harmonic regime is possible only under the resonant condition $\hbar\omega = U_0$. In the limit $\hbar\omega \gg k_b T$ the population transfer rate is

$$k_1 = V\hat{n}(\hbar\omega) = \frac{V}{e^{\beta\hbar\omega} - 1} \delta(\hbar\omega - U_0) \sim V e^{-\beta\hbar\omega} \delta(\hbar\omega - U_0) , \tag{40}$$

and therefore

$$\tau_{\text{Orb}} = \tau_0 e^{\beta U_{\text{eff}}} , \qquad \tau_0 = \frac{1}{V} , \qquad U_{\text{eff}} = \hbar\omega\delta(\hbar\omega - U_0) = U_0 , \tag{41}$$

where the usual identity $U_{\text{eff}} = U_0$ is recovered.

SUPPLEMENTARY NOTE 3. SPIN HAMILTONIAN PARAMETERS CALCULATION.

Let us consider the system to be described by an electronic Hamiltonian $\hat{H} = \hat{H}_{\mathrm{BO}} + \hat{H}_{\mathrm{SO}}$, where $\hat{H}_{\mathrm{SO}}$ refers to the spin-orbit component of the Hamiltonian and $\hat{H}_{\mathrm{BO}}$ refers to the Born-Oppenheimer one. The mapping between the electronic Hamiltonian, $\hat{H}$, and the spin Hamiltonian, $\hat{H}_{\mathrm{S}}$, is made through the relation

$$\langle SM_S|\hat{H}|SM'_S\rangle = \langle SM_S|\hat{H}_{\mathrm{S}}|SM'_S\rangle \, , \tag{42}$$

where $|SM_S\rangle$ are the spin eigenstate of total spin S and its z-component M_S. The $(2S{+}1)$ lower-lying eigenstates of $\hat{H}$ could be written as function of their eigenvalues and eigenvectors

$$\hat{H} = \sum_{k=1}^{2S+1} |k\rangle E_k \langle k| \, . \tag{43}$$

Therefore, the mapping with the spin Hamiltonian, $\hat{H}_{\mathrm{S}}$, assumes the form

$$\langle SM_S|\hat{H}_{\mathrm{S}}|SM_{S'}\rangle = \sum_{k}^{2S+1} \langle SM_S|k\rangle E_k \langle k|SM_{S'}\rangle \, . \tag{44}$$

Once E_k and $|k\rangle$ are obtained by means of electronic structure calculations, the system of linear equations (44) could be solved by means of a least square fit [8]. Although this relation is not *a priori* valid and the quality of the fitting should be evaluated each time, its range of applicability is wider with respect to the perturbation treatment of relativistic interactions [9]. For instance, it can also be exploited when higher order spin Hamiltonian terms are needed to describes the system; this feature is particularly appealing as analytical expressions are missing for these terms. At the same time, a clear connection between specific interactions included in $\hat{H}$ and spin Hamiltonian terms is here lost and the correctness of $\hat{H}_{\mathrm{S}}$ choice falls under the quality of the fit itself. Over parametrisation problems could arise, especially when multi spin complexes are considered. This procedure could be also applied to calculate the spin-phonon coupling coefficients in a complete general fashion. Considering an effective spin Hamiltonian $\hat{H}_{\mathrm{S}} = \sum_{kq} B_{kq}\hat{O}_{kq}(\vec{\mathbf{S}})$ [10], expressed as an expansion over even-order tesseral spin operators, $\hat{O}_{kq}$, the $\hat{H}_0$ matrix element derivatives could be evaluated as follows:

$$\sum_{i...j}\langle b|\frac{\partial^n \hat{H}_0}{\partial \hat{q}_i ... \partial \hat{q}_j}|a\rangle = \sum_{i...j}\frac{\partial^n}{\partial \hat{q}_i ... \partial \hat{q}_j}\langle b|\hat{H}_0|a\rangle = \sum_{i...j}\frac{\partial^n}{\partial \hat{q}_i ... \partial \hat{q}_k}\langle b|\hat{H}_{\mathrm{S}}|a\rangle = \sum_{i..j}\sum_{kq}\frac{\partial^n B_{kq}}{\partial \hat{q}_i ... \partial \hat{q}_j}\langle b|\hat{O}_{kq}(\vec{\mathbf{s}})|a\rangle \, . \tag{45}$$

In deriving Supplementary Eq. (45) we take advantage of the fact that the spin part of the wave-function does not depend on the bath normal modes, and of the spin hamiltonian definition $\langle b|\hat{H}_0|a\rangle = \langle b|\hat{H}_{\mathrm{S}}|a\rangle$. The expression (45) can be readily applied to any spin Hamiltonian form.

SUPPLEMENTARY NOTE 4. AB-INITIO CALCULATIONS DETAILS.

Spin dynamics calculations have been carried out for the anion $[(\mathrm{tpa}^{\mathrm{Ph}})\mathrm{Fe}]^-$ SMM [11], where $\mathrm{tpa}^{\mathrm{Ph}}$ = tris(pyrrolyl-phenyl-methyl)amine is the ligand of the $S = 2$ high-spin Fe^{2+} ion. The trigonal pyramidal coordination geometry of the amine ligands around the Fe^{2+} center makes the first excited $S = 2$ state almost degenerate with the ground state. This situation makes the spin-orbit interaction especially effective to split the degeneracy of $S = 2$ ground state multiplet, producing a remarkable easy axis anisotropy with a zero-field splitting of $D = -27.5$ cm^{-1} and with an almost vanishing rhombicity. $[(\mathrm{tpa}^{\mathrm{Ph}})\mathrm{Fe}]^-$ crystallizes in a $\mathrm{P}_{\bar{1}}$ space symmetry group with a primitive cell that comprises two SMMs units and two co-precipitate Na ion coordinated by three dimethoxyethane solvent molecules each, for a total of 228 atoms. The $[(\mathrm{tpa}^{\mathrm{Ph}})\mathrm{Fe}]^-$ structure has been optimized both for the isolated molecule and for the periodic unit cell in the gamma-point approximation. From now on these two models will be denoted as Isol Model and Bulk Model, respectively. The structures produced by the two optimisation schemes are very similar, as expected from the high stiffness of the ligands cage. Supplementary Table 1 reports the first coordination shell parameters for the X-ray experimental structure, the optimized Bulk Model structure and the optimized Isol Model one. The accuracy of the optimized Bulk Model internal degrees of freedom is remarkable as their deviation from the X-ray reference is about 1% or less.

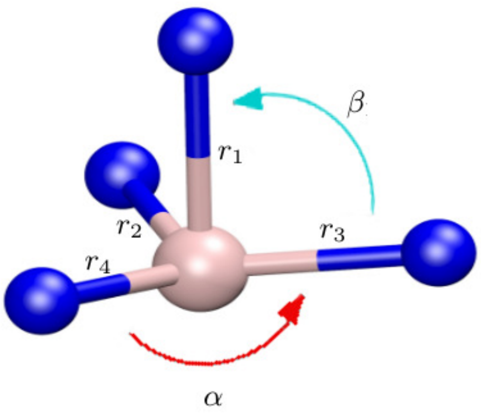

Supplementary Figure 2. **First Coordination Shell Structural Features Scheme.** Schematic representation of the Fe^{2+} (pink sphere) and N-ligands (blue sphere) geometry in $[(\mathrm{tpa}^{\mathrm{Ph}})\mathrm{Fe}]^-$. The symbols r_i describes the distances between the central Fe^{2+} ion and the first coordination shell N-ligands. The α and β labels represent the $\widehat{\mathrm{NFeN}}$ angles. α is used for the angles involving only N atoms in the plane perpendicular to the pseudo 3-fold symmetry axis while β is used for angles involving the N atom along the pseudo 3-fold symmetry axis.

Model	r_1 [b]	r_2	r_3	r_4	α_{23} [b]	α_{24}	α_{34}	β_{12} [b]	β_{13}	β_{14}
Exp [a]	2.161	2.013	2.019	2.016	120.22	115.56	120.27	83.84	83.37	82.88
Bulk	2.185	2.005	2.013	2.011	118.76	118.58	118.37	84.00	82.57	82.68
Isol	2.203	2.003	2.006	2.002	118.29	119.12	118.18	83.04	82.89	83.03

[a] Taken from Ref. [11].
[b] Distances are expressed in (Å) and angles in degrees.

Supplementary Table 1. **Principal internal structural parameters for the $[(\mathrm{tpa}^{\mathrm{Ph}})\mathrm{Fe}]^-$ models.** The experimental structural features (Exp Model) are compared with the periodic DFT (Bulk Model) and non-periodic DFT (Isol Model) optimized values. The symbols r_i describes the distances between the central Fe^{2+} ion and the first coordination shell N-ligands. The α and β labels represent the $\widehat{\mathrm{NFeN}}$ angles. α is used for the angles involving only N atoms in the plane perpendicular to the pseudo 3-fold symmetry axis while β is used for angles involving the N atom along the pseudo 3-fold symmetry axis.

The Bulk Model has been obtained through the optimization of both the ionic position inside the unit-cell and the lattice parameters. Supplementary Table 2 reports the comparison of the optimized lattice parameters with the experimental X-ray values. All the optimized lattice parameters show a very small deviation from the X-ray reference values, with errors of only a few percentage units. Moreover, it must be stressed that the X-ray structure has been collected at a temperature of 123 K [11], while our structures have been optimized at 0 K. Therefore, a shrinking of the simulated lattice parameters with respect to the X-ray reference is expected.

Normal modes and harmonic frequencies have been calculated for both the isolated and the periodic structure. The phonon density of states (DOSs) for both Isol Model and Bulk Model are displayed in Supplementary Fig. 3. Major differences between the two models are evident, especially in the low-energy part of the DOS. This clearly originates

Model	a (Å)	b (Å)	c (Å)	α (°)	β (°)	γ (°)	V (Å^3)
Exp[a]	11.360	12.028	17.881	76.897	73.871	63.850	2180.06
Bulk	11.265	11.687	17.014	78.812	76.177	72.096	2052.10

[a] Taken from Ref. [11].

Supplementary Table 2. **Lattice parameters for the [(tpa^{Ph})Fe]$^-$ models.** The X-ray experimental lattice parameters (Exp Model) are compared with the periodic DFT (Bulk Model) optimized values.

from the appearance of inter-molelcular vibrations. Interestingly, the Isol Model shows a non zero DOS at very low energy values. Further differences come from the presence in the periodic cell of the two Na^+ ions coordinated by three dimethoxyethanes each, which clearly contribute to the global DOS. Moreover, inter-molecular interactions are expected to introduce further modulation on the DOSs shape.

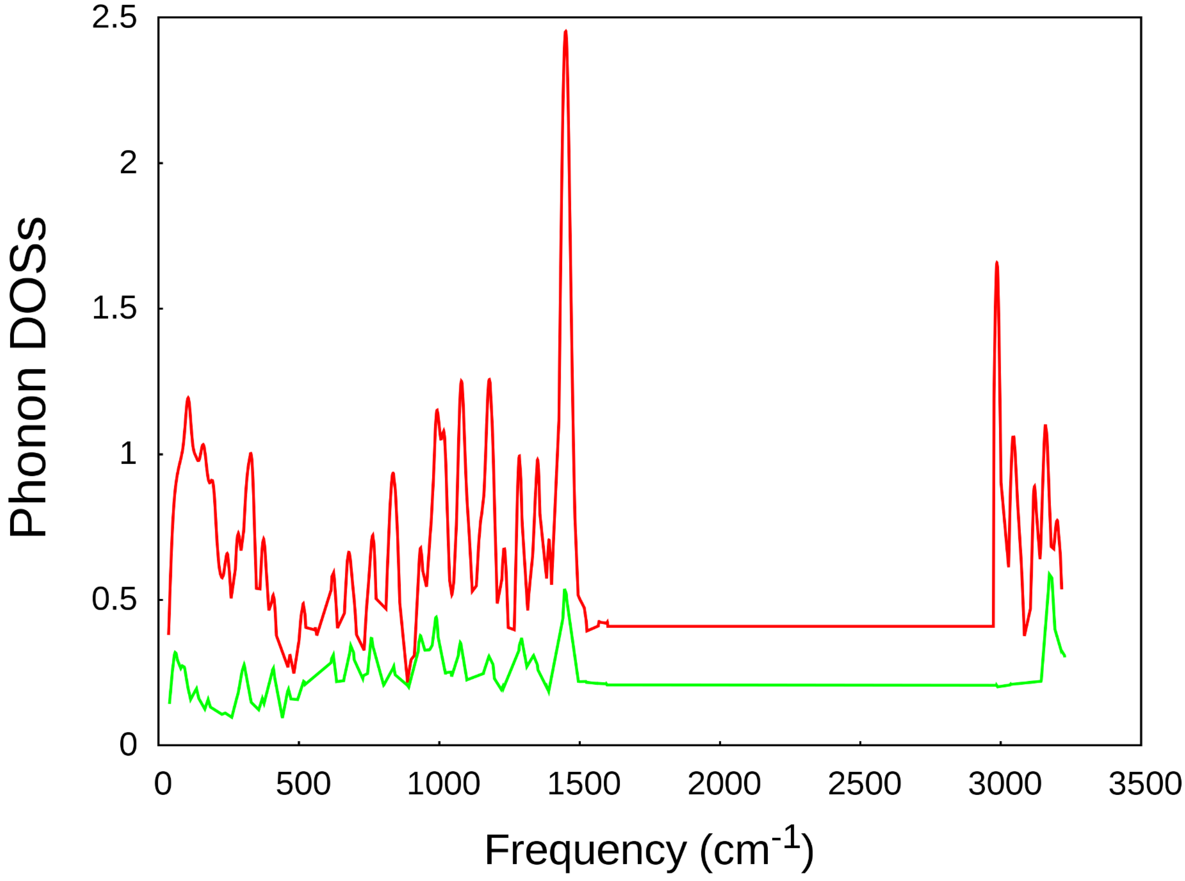

Supplementary Figure 3. **Calculated Phonon Density of States.** The calculated DOSs for the periodic DFT optimized model (Bulk Model) are reported with red lines and the calculated DOSs for the non-periodic DFT model (Isol Model) are reported with green lines. A gaussian broadening of 10 cm^{-1} has been applied to all the Γ-point frequencies to smoothen the plot.

CASSCF calculations have been performed in order to determine the [(tpa^{Ph})Fe]$^-$ zero-field splitting. The calculation of all the magnetic properties has been performed for a single isolated molecule as periodic boundary condition are not implemented for correlated calculations. For instance, for the Bulk Model only one of the two [(tpa^{Ph})Fe]$^-$ molecules in the primitive cell has been characterized. This does not represent a problem as they are symmetry-

related inside the crystal. However, the elimination of the explicit surrounding of the SMM during the anisotropy calculation poses a serious limitation to the model as the crystal is made of charged molecules. The calculation of magnetic properties in SMMs is usually carried for isolated molecules and, therefore, electrostatic long-range effects originating from the presence of the crystal environment are usually neglected. However, the importance of second coordination shell effects on the magnetic structure of SMMs has been reported [12]. In order to check if the system under study shows these kind of features we introduced a point charges model to describe the charge density of the periodic crystal.

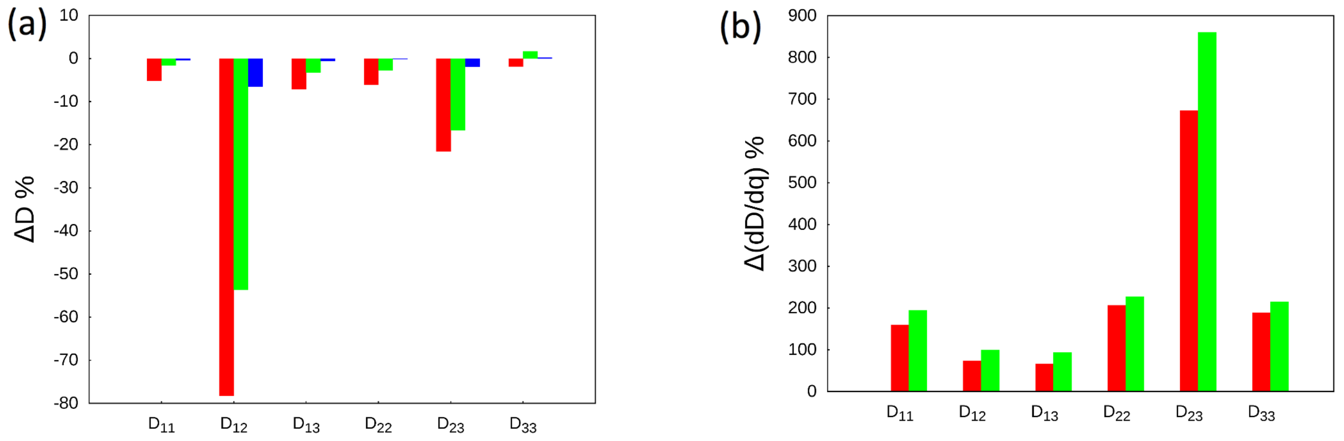

Supplementary Figure 4. **Stark effect on the calculated anisotropy tensor D.** Panel (a) shows the calculated **D** elements and their derivatives along an internal displacement (panel (b)) for $[(\mathrm{tpa}^{\mathrm{Ph}})\mathrm{Fe}]^{-}$. Data are expressed as percentage difference with respect to the isolated molecule results. Red bars show results for the calculation that includes the explicit nearest co-precipitate cation molecule. Blue bars show the results for the same model after the quenching of the co-precipitate charge through the removal of the Na^{+1} ions. Green bars show the results for the model with RESP point charges in place of the co-precipitate molecule.

Various point charge methods have been tested: Mulliken, density-derived point charges (DDAPC) [13], RESP [14] and CHELPG [15]. The first two methods rely on the projection of the DFT electronic structure on atomic localised functions, while the last two are based on a fitting of the electrostatic potential outside the van der Waals volume of the molecule coming from the DFT charge distribution. Although all these parametrisation schemes give similar results, final CASSCF calculations have been done with RESP point charges as this procedure is the one usually employed for force-field development and thus much more tested and robust.

Supplementary Fig. 4 shows results about the Stark effect on the zero-field split (ZFS) originating from charged co-precipitate molecules. Results coming from the introduction in the CASSCF simulation cell of one of the nearest co-precipitate molecule (red bars) show the dramatic impact of the Stark effect on both the ZFS and its derivatives, the last one being calculated along a low-energy internal vibrational mode. The substitution of the explicit co-precipitate molecule with its fitted RESP point charges reproduces very nicely the results. It also must be stressed out that the explicit molecule model is not necessarily the most accurate one. Indeed the co-precipitate charged molecules is treated at the HF theory level, which is known to overestimate both the dipole moment and the polarisability [16]. Accordingly, DFT GGA fitted RESP point charges predict a smaller Stark effect. Calculations with the explicit co-precipitate molecule without the Na ions have also been carried out. The quenching of the net charge shows that dipole and polarisability effects have only a small impact on the magnetism of the SMM. In order to mimic the periodic surrounding, the single explicit SMM has been embedded in a $21\times21\times21$ lattice of point charges. The model that includes the point charge correction is called Bulk PC Model. This model shares the same structure and phonon structure of the Bulk Model.

Before proceeding to the discussion of the **D** tensor calculations, a comment on the validity of the spin Hamiltonian itself is required. In a completely general fashion, the spin Hamiltonian formalism can be applied to describe the splitting of the spin energy levels as long as the wave-functions of the electronic states considered [see Supplementary Eq. (43)] possesses a well-defined S^2 expectation value, *i.e.* there is no spin contamination. Technically this becomes an issue only after the inclusion of the spin-orbit interaction, which may lead to a ground state multiplet with non-vanishing orbital angular momentum. In general Jahn-Teller activity removes the problem, as in the present case, where the global $\mathbf{C}_3$ molecular symmetry is relaxed to a lower $\mathbf{C}_1$ symmetry, as can also be seen from Supplementary Table 1. However, the stiffness of the first coordination shell makes the Jahn-Teller distortions not fully effective in removing the degeneracy of the first two excited states and the final low-energy-lying spin-orbit-corrected wave-

functions are indeed a superposition of the two different eigenstates of the Born-Oppenheimer Hamiltonian. However, in this case the two solutions have both an S=2 multiplicity and, therefore, this situation does not impose any formal restriction on the use of the spin Hamiltonian. It must also be stressed that the spin Hamiltonian formalism has also been already used before to study $[(\mathrm{tpa^{Ph}})\mathrm{Fe}]^-$ and other synthetic analogs [11, 17]. Moreover, it is worth mentioning that our framework does not necessarily involve the use of the spin Hamiltonian. Indeed, one can directly use the expression (43) to evaluate the matrix elements in the first term of Supplementary Eq. (45), instead of passing by the fitting of the spin Hamiltonian. Supplementary Table 3 reports the calculated anisotropy parameters for all the models equilibrium structures.

	Exp[a]	Models			
		X-ray	Isol	Bulk	Bulk-PC
D ($\mathrm{cm^{-1}}$)	-27.5	-27.54	-33.75	-30.74	-31.01
E/D	0.08	0.03	0.01	0.05	0.06

[a] Taken from [11]

Supplementary Table 3. **Calculated Anisotropy constant D and rhombic parameter E for the $[(\mathrm{tpa^{Ph}})\mathrm{Fe}]^-$ models.** X-ray refears to the calculation done with the experimental X-ray structure, Isol refears to the calculation done with the $[(\mathrm{tpa^{Ph}})\mathrm{Fe}]^-$ DFT optimized structure in vacuum and Bulk refears to the calculation done with the $[(\mathrm{tpa^{Ph}})\mathrm{Fe}]^-$ periodic-DFT optimized structure. Bulk-PC refears to a model sharing the same Bulk model structure but with the addiction of a 21x21x21 point charges lattice mimicking the periodic crystal electric field.

The D axial anisotropy constant calculated retaining the X-ray structure (X-ray model), without any optimisation, is found very close to the experimental one demonstrating the ability of CASSCF to handle this problem, as also previously noted by Atanasov *et al.* [17]. By looking at the various models it is possible to observe a small deviation from the X-ray value. Bulk Model is the one, which better reproduces the experimental value, according to its higher level of environment quality. Interestingly, the effect of the point charge corrections is almost negligible. Although preliminary calculations predict an important effect of an externally applied electrostatic field on the anisotropy, here the local field is everywhere zero due to the crystal symmetry and thus ineffective.

Although the calculated axial anisotropy parameters are in good agreement with the experimental values, the reproduction of the entire ground state $S = 2$ multiplet spectrum is less satisfactory. Indeed, energy levels of the second pseudo doublet, corresponding to $DS^2 - D(S-1)^2 = D(2^2 - 1^2) = -3D$, are overestimated by about 15 $\mathrm{cm^{-1}}$ with respect to the CASSCF calculated ones, while the energy of the highest energy spin level,corresponding to $DS^2 - D(S-2)^2 = D(2^2 - 0^2) = -4D$, is underestimated by about the same amount. The breaking down of the fit to a spin Hamiltonian containing only second order Stevens terms is in line with the high anisotropy of the molecule. Indeed, the large ZFS observed for this SMM originates from the presence of two almost degenerate electronic energy levels with the same spin multiplicity, which mix to create the $S = 2$ ground state spin multiplet once the SO interaction is turned on[17]. However, as the ground state multiplet does not suffer from S-mixing effects the quality of its description could be improved by employing higher-order terms in the spin Hamiltonian. Indeed, tests along this direction showed an increased quality of the CASSCF spin levels fitting when fourth order terms $\hat{O}_{kq}(\vec{\mathbf{S}})$ are added to the spin Hamiltonian. However, the introduction of these terms does not change the results discussed in the next sections, except for an overall speeding up of the relaxation process thanks to new spin-phonon coupling terms. For this reason all the discussion on the spin dynamics is done over the data obtained with a spin Hamiltonian $\hat{H}_{\mathrm{S}} = \sum_{ij} D_{ij}\hat{S}_i\hat{S}_j$ without any loss of generality.

First and second order derivatives of the $\mathbf{D}$ tensor, with respect to the phonon variables, have been computed numerically by finite differences with a step (δq_α) of 0.2 normal mode units, according to the relations:

$$\frac{\partial D_{ij}}{\partial q_\alpha} = \frac{D_{ij}(\delta q_\alpha) - D_{ij}(-\delta q_\alpha)}{2\delta q_\alpha}, \quad \frac{\partial^2 D_{ij}}{\partial q_\alpha^2} = \frac{D_{ij}(\delta q_\alpha) + D_{ij}(-\delta q_\alpha) - 2D_{ij}}{\delta q_\alpha^2}, \tag{46}$$

where D_{ij} and $D_{ij}(\pm\delta q_\alpha)$ represents the ij element of the $\mathbf{D}$ tensor at the equilibrium geometry and at the distorted one, respectively. These two derivatives have also been extracted by interpolation of D_{ij} elements as function of the displacement with a quadratic function $f_{ij}(q_\alpha) = \frac{1}{2}\frac{\partial^2 D_{ij}}{\partial q_\alpha^2} q_\alpha^2 + \frac{\partial D_{ij}}{\partial q_\alpha} q_\alpha + k$, where k is just an offset constant. The numerical value of both first and the second order derivative of the D_{ij} elements, calculated with the two different

methods, coincides for all the available digits for each phonon, assessing the quality of the calculated parameters. Supplementary Fig. 5 reports the D_{ij} values as function of the displacement q_α for the first normal mode (36 cm^{-1}) of the Bulk Model and the corresponding fitted $f_{ij}(q_\alpha)$ functions.

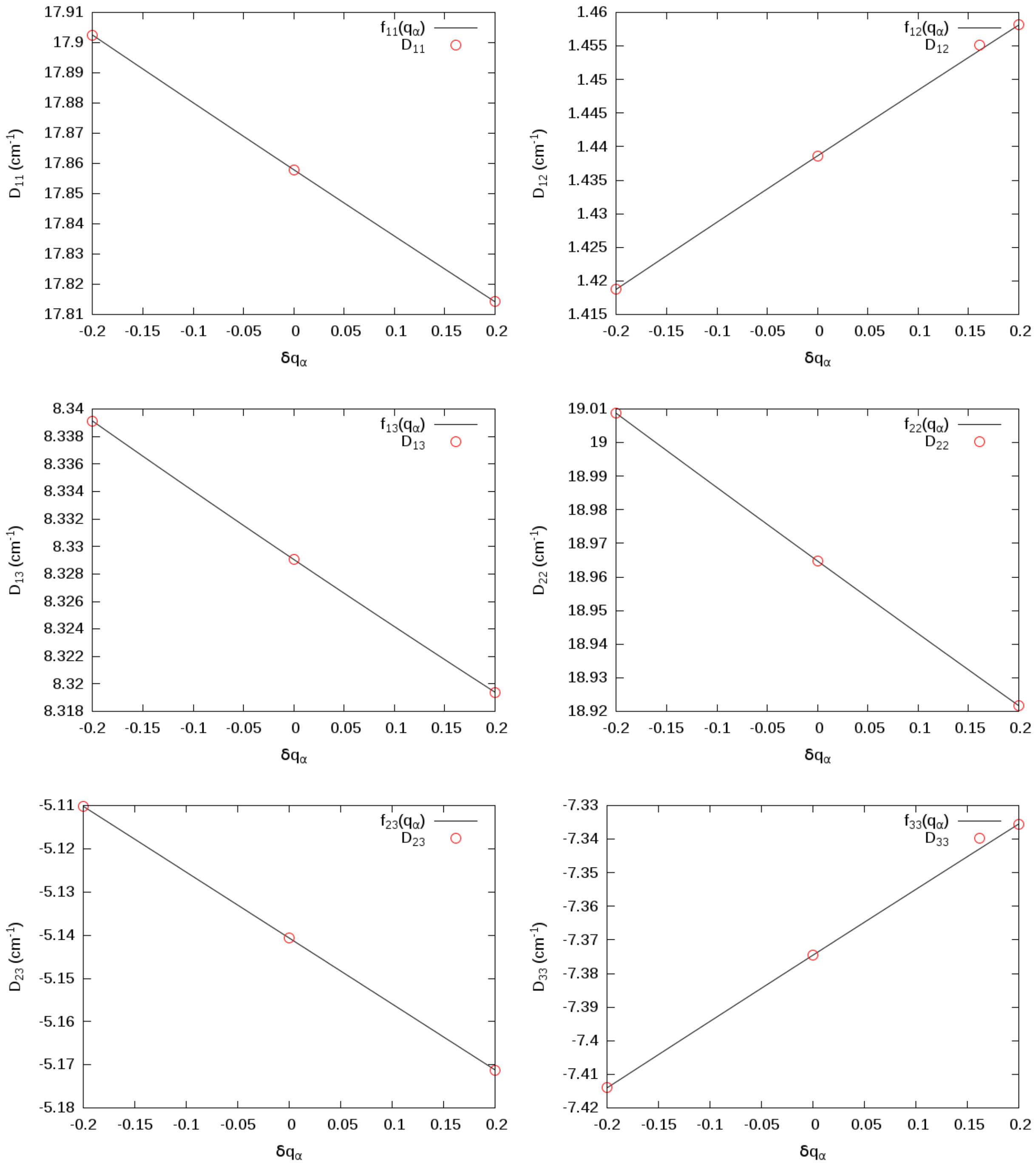

Supplementary Figure 5. **D tensor dependence on nuclear displacements.** The red open circles represent the value of the anisotropy tensor elements D_{ij} as function of the q_α normal mode displacement at frequency 36 cm^{-1}. The solid black lines correspond to the fitted $f_{ij}(q_\alpha) = \frac{1}{2}\frac{\partial^2 D_{ij}}{\partial q_\alpha^2} q_\alpha^2 + \frac{\partial D_{ij}}{\partial q_\alpha} q_\alpha + k$ functions for each D_{ij}.

The best fitted values obtained from the optimization of the $f_{ij}(q_\alpha)$ functions, corresponding to the first and second order derivatives of the D_{ij} parameters with respect to the q_α normal mode with vibrational frequency 36 cm^{-1}, are reported in Supplementary Table 4 The determination of the second order derivatives of **D** with respect to a single

**D** Element	11	12	13	22	23	33
$\frac{\partial D_{ij}}{\partial q_\alpha}$ (cm^{-1})	-0.221	0.098	-0.049	-0.218	-0.152	0.196
$\frac{\partial^2 D_{ij}}{\partial q_\alpha^2}$ (cm^{-1})	0.021	-0.010	0.010	0.022	-0.001	-0.011

Supplementary Table 4. **Fitted Spin-Phonon coupling coefficients.** Numerical Derivatives of the anisotropy tensor **D** along the Normal Mode at 36 cm^{-1} obtained by finite difference displacements.

normal mode $\hat{q}_\alpha$ also offers the possibility to calculate the average effect of the phonon bath dynamics on the spin Hamiltonian. This effect has been proposed by Pederson et al. [18] and very recently by Moreno et al. [19] to be at the origin of a spin Hamiltonian parameters renormalization effect. By assuming the spin-phonon coupling Hamiltonian to be

$$\hat{H}_{\mathrm{s-ph}} = \sum_\alpha \left[\frac{\partial \hat{H}_{\mathrm{S}}}{\partial \hat{q}_\alpha} + \frac{1}{2} \frac{\partial^2 \hat{H}_{\mathrm{S}}}{\partial \hat{q}_\alpha^2} \right] , \tag{47}$$

it is possible to evaluate the renormalization of the spin Hamiltonian parameters due to the average effect of the bath and defining a new $\hat{\bar{H}}_{\mathrm{S}}$ as

$$\hat{\bar{H}}_{\mathrm{S}} = \hat{H}_{\mathrm{S}} + \mathrm{Tr}_B[\hat{H}_{\mathrm{s-ph}} \hat{\rho}_B] \, . \tag{48}$$

Only the second order derivatives of the spin Hamiltonian contribute to the trace over the bath degrees of freedom appearing in Supplementary Eq. (48), leading to

$$\hat{\bar{H}}_{\mathrm{S}} = \hat{H}_{\mathrm{S}} + \sum_\alpha \frac{\partial^2 \hat{H}_{\mathrm{S}}}{\partial \hat{q}_\alpha^2} \left(\bar{n}_\alpha + \frac{1}{2} \right) . \tag{49}$$

According to Supplementary Table 4 and Supplementary Eq. (49) it is possible to estimate for the mode $\omega = 36$ cm^{-1} a phonon-induced modification of the D_{ij} elements of the order of a fraction of cm^{-1} in the investigated temperature range. This is clearly only a negligible correction to the effects discussed in the paper and can not be assumed to be at the origin of the massive spin-flip energy barrier reductions observed in literature.

SUPPLEMENTARY NOTE 5. MAGNETIZATION DYNAMICS.

The magnetisation dynamics has been studied by solving the Redfield equation for the diagonal elements of the spin reduced density matrix:

$$\frac{d\rho^{\mathrm{S}}_{aa}(t)}{dt} = \frac{2}{\hbar^2}\sum_c\sum_\alpha \mathcal{M}^\alpha_{ac}\rho^s_{cc}(t)\ , \quad \mathcal{M}^\alpha_{ac} = -\sum_j V^\alpha_{aj}V^\alpha_{jc}\delta_{ac}G(\omega_{jc},\omega_\alpha) + |V^\alpha_{ac}|^2 G(\omega_{ca},\omega_\alpha)\ , \tag{50}$$

where $\rho^{\mathrm{S}}_{ab} = \langle a|\hat{\rho}^{\mathrm{S}}|b\rangle$, $V^\alpha_{ab} = \langle a|\frac{\partial \hat{H}_{\mathrm{S}}}{\partial \hat{q}_\alpha}|b\rangle$, $|a\rangle$ is the eigenfunction of the spin system described by the Hamiltonian H_{S} with energy E_a and $\omega_{ab} = (E_a - E_b)/\hbar$. Thus in Supplementary Eq. (50) $|V^\alpha_{ac}|^2 G(\omega_{ca},\omega_\alpha)$ represents the kinetic rate of population transfer between the eigenstates $|a\rangle$ and $|c\rangle$.

From Supplementary Eq. (50) it is possible to calculate the evolution in time of the eigenstates population, which in turn can be used to compute the evolution in time of the magnetization expectation value as

$$\langle \vec{\mathbf{M}}(t)\rangle = \sum_a \langle a|\hat{\rho}^{\mathrm{S}}(t)\vec{\mathbf{M}}|a\rangle\ . \tag{51}$$

If we now choose of solving only the diagonal part of the Redfield Eqs. (50), we will restrict our study to the dynamics of pure states. In this case, the starting spin density matrix $\hat{\rho}^{\mathrm{S}}(t=0)$ must be built from one of the eigenvectors of the spin Hamiltonian $\hat{H}_{\mathrm{S}}$ in order for the off-diagonal terms of $\hat{\rho}^{\mathrm{S}}$ to be zero at all times. The results displayed in the main text has been obtained by choosing $\hat{\rho}^{\mathrm{S}}(t=0) = |0\rangle\langle 0|$, where $|0\rangle$ corresponds to the lowest in energy eigenket of $\hat{H}_{\mathrm{S}}$. One can visualize this *in silico* experiment as the preparation of the quantum state by thermalizing the system at a temperature where only the ground state is populated and its afterward quench by abruptly increasing the temperature to the desired value. The evolution in time of the z-component of $\vec{\mathbf{M}}$ is showed by the red line in Supplementary Fig. 6. The expectation value of M_z at $t = 0$ is slightly lower than 2 due to the presence of the rhombic term in $\hat{H}_{\mathrm{S}}$, which mixes the $\hat{S}_z$ components in the $\hat{H}_{\mathrm{S}}$ eigenkets and lowers the magnetization expectation value. This effect is reduced by the presence of the external magnetic field (1500 Oe) present in the spin Hamiltonian but it can never be completely cancelled out. The time profile of M_z is purely exponential and it has been used to extract the relaxation time, τ, from the fitting of $M_z(t) = (M_z(0) - M_z(\infty))e^{-t/\tau} + M_z(\infty)$. In order to investigate the dependence of the spin relaxation time from the starting condition we also solved the full Redfield equation (52). This equation describes the evolution in time of all the $\hat{\rho}^{\mathrm{S}}$ matrix elements and makes possible to study the evolution of arbitrary starting states.

$$\frac{d\rho^{\mathrm{S}}_{ab}(t)}{dt} = \frac{1}{\hbar^2}\sum_{a,b,c,d}\sum_\alpha \mathcal{R}^\alpha_{ab,cd}\rho^{\mathrm{S}}_{cd}(t)\ , \tag{52}$$

where

$$\mathcal{R}^\alpha_{ab,cd} = -\sum_j \delta_{bd}V^\alpha_{aj}V^\alpha_{jc}G(\omega_{jc},\omega_\alpha) - \sum_j \delta_{ca}V^\alpha_{dj}V^\alpha_{jb}G(\omega_{jd},\omega_\alpha) + V^\alpha_{ac}V^\alpha_{db}G(\omega_{db},\omega_\alpha) + V^\alpha_{ac}V^\alpha_{db}G(\omega_{ca},\omega_\alpha)\ . \tag{53}$$

By Considering the dynamics for only the diagonal part of $\hat{\rho}^{\mathrm{S}}$ one gets back the expression in Supplementary Eq. (50), *i.e.* $\mathcal{R}^\alpha_{aa,bb} \rightarrow 2\mathcal{M}^\alpha_{a,b}$. The blue line of Supplementary Fig. 6 displays the dynamics of M_z when the density matrix is initialised as pure maximally polarised $\hat{S}_z$ eigenstate. This scenario can be obtained in experiments by a preparation of the quantum state in an extremely high external magnetic field B followed by a quench due to the reduction of B to the desired value.

In this situation the exponential decay is superimposed to an oscillating function arising from the phase dynamics. This fast ($\sim$ THz) wave function oscillations are the analog of a particle-through-barrier tunnelling effect. Indeed, if a generic quantum system is not initialised as an eigenstate, it will oscillate coherently between its eigenstates or part of them. This is exactly what happens to the spin system as long as it is not interacting with a bath. Clearly such oscillations are not part of a relaxation mechanism as the phase state dynamics is not damped. As soon as the interaction with a bath is switched on, every elements of the density matrix will start decaying. This effect must not be confused with the so called quantum tunnelling relaxation, which is instead a direct relaxation mechanism between two degenerate H_{S} eigenstates due to inter molecule dipolar interactions. Quantum tunnelling relaxation is not covered by our simulations as only one SMM has been considered.

Even though the starting magnetisation is different in the two cases, the exponential-decay characteristic time is the same. This happens because the nature of the equilibrium driving force is unchanged in the two experiments and the population decaying part of the equation remains the same. For this reason all the calculations have been done by utilising only the diagonal elements of Supplementary Eq. (52), without any loss of generality.

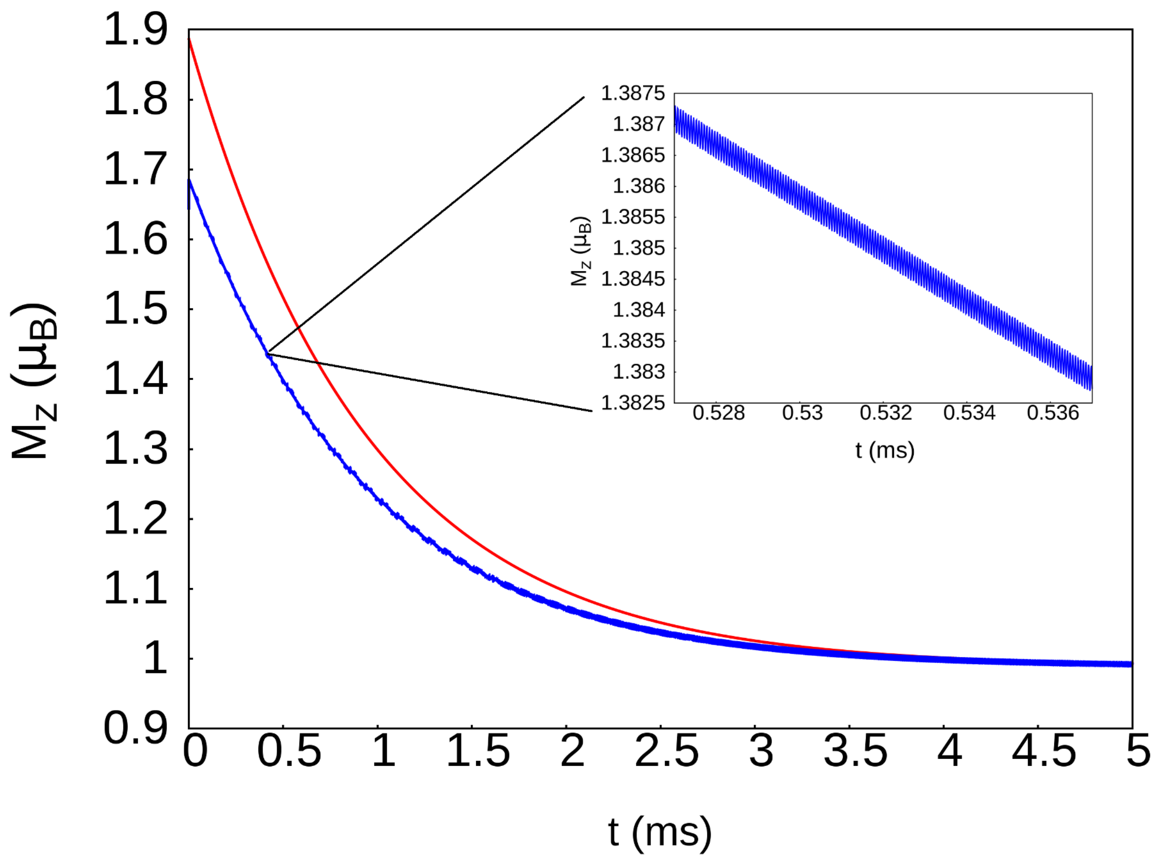

Supplementary Figure 6. **Time dependence of the magnetization z-component expectation value at 5 K.** The red line corresponds to the dynamics of the system initialized as an eigenstate of $\hat{H}_S$, while the blue one corresponds to the dynamics of the system initialized as an eigenstate of $\hat{S}_z$. The inset shows the Rabi oscillations occurring in the latter case.

SUPPLEMENTARY NOTE 6. CP2K INPUT.

Here it follows the input file it has been used for periodic-DFT calculations with the software cp2k. As output of the vibrational analysis kind of calculation one gets frequencies and normal modes of the simulation cell in the molden format. Starting from these values is it possible to calculate the cartesian displacements associated to each normal mode and use them to perturb the Γ-point optimized geometry of a given molecule. This new set of distorted geometries has then be used as input for the ORCA software for the magnetic properties calculation.

```
&GLOBAL
  PRINT_LEVEL  MEDIUM
  PROJECT_NAME <name>
  RUN_TYPE  <VIBRATIONAL_ANALYSIS or GEO_OPT>
  FLUSH_SHOULD_FLUSH  T
&END GLOBAL

&VIBRATIONAL_ANALYSIS
 &PRINT
  &MOLDEN_VIB
   FILENAME <file-name>
  &END
 &END
 DX 0.001
 FULLY_PERIODIC TRUE
&END

&MOTION
  &GEO_OPT
    MAX_DR     1.0000000000000003E-04
    MAX_FORCE     9.9999999999999974E-06
  &END GEO_OPT
  &CELL_OPT
    MAX_DR     1.0000000000000003E-04
    MAX_FORCE     9.9999999999999940E-06
    TYPE  DIRECT_CELL_OPT
  &END CELL_OPT
  &PRINT
    &TRAJECTORY  SILENT
      FILENAME <file-name>
    &END TRAJECTORY
    &RESTART  SILENT
      FILENAME <file-name>
    &END RESTART
  &END PRINT
&END MOTION

&FORCE_EVAL
  METHOD  QS
  &DFT
    BASIS_SET_FILE_NAME <file-name>
    POTENTIAL_FILE_NAME <file-name>
    WFN_RESTART_FILE_NAME <file-name>
    UKS  T
    MULTIPLICITY 9
    &SCF
      MAX_SCF  40
      EPS_SCF     5.0000000000000004E-08
      CHOLESKY  INVERSE
      SCF_GUESS  RESTART
```

```
      ADDED_MOS  500
      &SMEAR  T
        METHOD  FERMI_DIRAC
        ELECTRONIC_TEMPERATURE     3.5000000000000000E+03
      &END SMEAR
      &MIXING  T
        METHOD  BROYDEN_MIXING
        ALPHA     1.0000000000000001E-01
        BETA     1.5000000000000000E+00
        NBUFFER  8
      &END MIXING
      &PRINT
        &RESTART  SILENT
          ADD_LAST  NUMERIC
          FILENAME <file-name>
        &END RESTART
      &END PRINT
    &END SCF
    &QS
      EXTRAPOLATION  ASPC
      EXTRAPOLATION_ORDER  1
    &END QS
    &MGRID
      NGRIDS  5
      CUTOFF     6.0000000000000000E+02
      REL_CUTOFF     5.5000000000000000E+01
    &END MGRID
    &XC
      DENSITY_CUTOFF     1.0000000000000000E-10
      GRADIENT_CUTOFF     1.0000000000000000E-10
      TAU_CUTOFF     1.0000000000000000E-10
      &XC_GRID
        XC_SMOOTH_RHO  NN50
        XC_DERIV  NN50_SMOOTH
      &END XC_GRID
      &XC_FUNCTIONAL  NO_SHORTCUT
        &PBE  T
        &END PBE
      &END XC_FUNCTIONAL
      &VDW_POTENTIAL
        POTENTIAL_TYPE  PAIR_POTENTIAL
        &PAIR_POTENTIAL
          TYPE  DFTD3
          PARAMETER_FILE_NAME <file-name>
          REFERENCE_FUNCTIONAL PBE
        &END PAIR_POTENTIAL
      &END VDW_POTENTIAL
    &END XC
  &END DFT
  &SUBSYS
    &CELL
      A     1.1265091075329234E+01    0.0000000000000000E+00    0.0000000000000000E+00
      B     3.5929899509396002E+00    1.1121411890678370E+01    0.0000000000000000E+00
      C     4.0649156911286246E+00    2.1560074656255934E+00    1.6379649398484094E+01
      MULTIPLE_UNIT_CELL  1 1 1
    &END CELL
    &KIND H
      BASIS_SET DZVP-MOLOPT-SR-GTH
```

```
      POTENTIAL GTH-PBE-q1
    &END KIND
    &KIND Na
      BASIS_SET DZVP-MOLOPT-SR-GTH
      POTENTIAL GTH-PBE-q9
    &END KIND
    &KIND C
      BASIS_SET DZVP-MOLOPT-SR-GTH
      POTENTIAL GTH-PBE-q4
    &END KIND
    &KIND N
      BASIS_SET DZVP-MOLOPT-SR-GTH
      POTENTIAL GTH-PBE-q5
    &END KIND
    &KIND O
      BASIS_SET DZVP-MOLOPT-SR-GTH
      POTENTIAL GTH-PBE-q6
    &END KIND
    &KIND Fe
      BASIS_SET DZVP-MOLOPT-SR-GTH
      ELEMENT Fe
      POTENTIAL GTH-PBE-q16
      &BS  T
        &ALPHA
          NEL  -2 -1
          L  0 2
          N  4 3
        &END ALPHA
        &BETA
          NEL  -2 -5
          L  0 2
          N  4 3
        &END BETA
      &END BS
    &END KIND
    &TOPOLOGY
      COORD_FILE_NAME <file-name>
      COORD_FILE_FORMAT  XYZ
      NUMBER_OF_ATOMS  228
      MULTIPLE_UNIT_CELL  1 1 1
    &END TOPOLOGY
  &END SUBSYS
&END FORCE_EVAL
```

SUPPLEMENTARY REFERENCES

[1] S. Califano, V. Schettino, and N. Neto, *Lattice dynamics of molecular crystals*, Vol. 26 (Springer Science & Business Media, 2012).

[2] M. Rigol, V. Dunjko, and M. Olshanii, "Thermalization and its mechanism for generic isolated quantum systems," Nature, **452**, 854–858, (2008).

[3] R. Kubo, "Stochastic Liouville Equations," J. Math. Phys., **4**, 174–183, (1963).

[4] R. Kubo and K.E. Shuler, "Stochastic processes in chemical physics," Advances in Chemical Physics, **15**, 101, (1969).

[5] R. Torre, R. Righini, L. Angeloni, and S. Califano, "Picosecond measurements of relaxation of internal modes in crystalline benzene as a function of temperature," J. Chem. Phys., **93**, 2967–2973, (1990).

[6] R. Bini, S. Califano, B. Eckert, and H. J. Jodl, "Temperature dependence of the vibrational relaxation processes in natural and isotopically pure 32S8: Effect of the isotopic impurities on infrared phonon lifetimes," J. Chem. Phys., **106**, 511–518, (1997).

[7] J. P. Pinan, R. Ouillon, P. Ranson, M. Becucci, and S. Califano, "High resolution Raman study of phonon and vibron bandwidths in isotopically pure and natural benzene crystal," J. Chem. Phys., **109**, 5469–5480, (1998).

[8] R Maurice, R Bastardis, C Graaf, N Suaud, T Mallah, and N Guihéry, "Universal theoretical approach to extract anisotropic spin Hamiltonians," J. Chem. Theory Comput., **5**, 2977–2984, (2009).

[9] F. Neese and E.I. Solomon, "Calculation of Zero-Field Splittings, g-Values, and the Relativistic Nephelauxetic Effect in Transition Metal Complexes. Application to High-Spin Ferric Complexes." Inorg. Chem., **37**, 6568–6582, (1998).

[10] W.C. Tennant and C.J. Walsby, "Rotation matrix elements and further decomposition functions of two-vector tesseral spherical tensor operators; their uses in electron paramagnetic resonance spectroscopy," J. Phys. Condens. Matter, **12**, 9481–9495, (2000).

[11] W.H. Harman, T.D. Harris, D.E. Freedman, H. Fong, A. Chang, T.D. Rinehart, Andrew Ozarowski, M.T. Sourgrati, F. Grandjean, J.G. Long, J.R. Long, and C.J. Chang, "Slow magnetic relaxation in a family of trigonal pyramidal iron (II) pyrrolide complexes," J. Am. Chem. Soc., **132**, 18115–18126, (2010).

[12] G. Cucinotta, M. Perfetti, J. Luzon, M. Etienne, P-E. Car, A. Caneschi, G. Calvez, K. Bernot, and R. Sessoli, "Magnetic anisotropy in a dysprosium/DOTA single-molecule magnet: beyond simple magneto-structural correlations." Angew. Chem. Int. Ed. Engl., **51**, 1606–10, (2012).

[13] P.E. Blöchl, "Electrostatic decoupling of periodic images of plane-wave-expanded densities and derived atomic point charges," J. Chem. Phys., **103**, 7422–7428, (1995).

[14] C.I. Bayly, P. Cieplak, W. Cornell, and Peter A Kollman, "A well-behaved electrostatic potential based method using charge restraints for deriving atomic charges: the resp model," J. Phys. Chem., **97**, 10269–10280, (1993).

[15] C.M. Breneman and K.B. Wiberg, "Determining atom-centered monopoles from molecular electrostatic potentials. the need for high sampling density in formamide conformational analysis," J. Comput. Chem., **11**, 361–373, (1990).

[16] A.L. Hickey and C.N. Rowley, "Benchmarking quantum chemical methods for the calculation of molecular dipole moments and polarizabilities." J. Phys. Chem. A, **118**, 3678–87, (2014).

[17] M. Atanasov, D. Ganyushin, D. A Pantazis, K. Sivalingam, and F. Neese, "Detailed ab initio first-principles study of the magnetic anisotropy in a family of trigonal pyramidal iron (II) pyrrolide complexes." Inorg. Chem., **50**, 7460–7477, (2011).

[18] M.R. Pederson, N. Bernstein, J. Kortus, "Forth-order magnetic anisotropy and tunnel splittings in Mn_{12} from spin-orbit-vibron interactions." Phys. Rev. Lett., **89**, 097292–097296, (2002).

[19] L. Escalera Moreno, N. Suaud, A. Gaita Arino, "Theoretical determination of the spin-vibration coupling in the highly coherent molecular spin qubit $[Cu(mnt)_2]^{2-}$." Preprint at: http://arxiv.org/abs/1512.05690, (2016).
